# Effects of arbuscular mycorrhizal fungi on flavonoid content in *Astragali Radix* cultivated in cadmium-contaminated soils

**DOI:** 10.3389/fmicb.2025.1576236

**Published:** 2025-04-24

**Authors:** Xiu-xin Meng, Xia Jia, Yong-hua Zhao

**Affiliations:** ^1^Key Laboratory of Subsurface Hydrology and Ecological Effects in Arid Region of Ministry of Education, Shaanxi Key Laboratory of Land Consolidation, School of Water and Environment, Chang’an University, Xi’an, China; ^2^School of Land Engineering, Chang’an University, Xi’an, China

**Keywords:** *Funneliformis mosseae*, flavonoid monomers, cadmium, phenylalanine ammonia-lyase gene, chalcone synthase gene

## Abstract

**Introduction:**

As bioactive components in *Astragali Radix* (AR), flavonoids can promote hematopoiesis and have hypolipidemic properties, among others, and they are easily affected by environmental factors. Arbuscular mycorrhizal fungi (AMF) can influence flavonoid synthesis in plants exposed to heavy metals by expanding the root absorption area to establish a reciprocal symbiotic relationship with most plants.

**Methods:**

We investigated the effects of *Funneliformis mosseae* and time on the total flavonoids and key monomers (calycosin, calycosin-7-glucoside, formononetin, and ononin) in AR exposed to cadmium (Cd) using a pot experiment. The treatments consisted of non-inoculation and *F. mosseae* inoculation. A two-way analysis of variance and Duncan’s test were conducted.

**Results:**

Shoot total flavonoids decreased (*p* < 0.05) at 60 (20.5%) and 90 d (18.3%), while formononetin decreased (83.4%) by 120 d; conversely, calycosin-7-glucoside increased (*p* < 0.05) with inoculation, although calycosin-7-glucoside decreased (*p* < 0.05) over time from 60 to 120 d regardless of inoculation. Shoot calycosin increased (*p* < 0.05) over time regardless of inoculation. Root total flavonoids decreased (*p* < 0.05) by 15.2% at 60 d, then increased (*p* < 0.05) by 23.5% at 90 d, along with increases in formononetin (117.1%) and ononin (59.6%) at 60 d, and calycosin-7-glucoside (21.2%) at 120 d, which increased (*p* < 0.05) under inoculation. The colonization rate, along with shoot Cd, C, P, H, and C/N ratio, significantly affected shoot flavonoids, while Cd accounted for 90.0% of flavonoid variation, which may be associated with its impact on flavonoid synthase. The variation in root flavonoids was significantly influenced by root S, biomass, and N, suggesting that AMF regulation may vary between AR organs. Calycosin-7-glucoside was significantly affected by phenylalanine ammonia-lyase (a key gene in flavonoid synthesis). Overall, *F. mosseae* led to significant increases in shoot total flavonoids and calycosin-7-glucoside. The total flavonoids were higher in shoots than in roots, indicating that annual AR shoots exposed to Cd may be utilized for medicinal purposes under inoculation.

**Discussion:**

These results provide insights into the enhancement of AMF on the quality of medicinal plants grown in Cd-contaminated soils, and the long-term effects of AMF on flavonoids at varying Cd levels should be further investigated.

## Introduction

As an important medicinal plant, *Astragali Radix* (AR) is known to strengthen the spleen, nourish the blood, stabilize the body’s surface, enhance immunity, promote hematopoiesis, exhibit hypolipidemic effects, and regulate blood pressure due to its rich content of flavonoids, saponins, and polysaccharides (Kondeva-Burdina et al., 2019; Chen et al., 2020; Zhang et al., 2021; Wang et al., 2023a). However, the synthesis and accumulation of flavonoids in plants are easily affected by various factors, such as soil microbes and heavy metals. Some studies have shown that low levels of heavy metals, such as cadmium (Cd) and aluminum, can significantly stimulate the synthesis of flavonoids in certain plants, including *Hypoxis hemerocallidea* Fisch. & C.A. Mey, *Robinia pseudoacacia* L., and *Triticum aestivum L.* (Okem et al., 2015; Jia et al., 2014; Zhang et al., 2023).

The drainage of industrial waste, application of chemical fertilizers, sewage irrigation, and mining have led to heavy metal pollution in the soil, creating a significant environmental issue. As a highly persistent and toxic heavy metal, cadmium (Cd) poses a significant threat to plant growth and metabolism (Paz-Ferreiro et al., 2017). Therefore, Cd-contaminated soils require adequate attention. The large-scale synthesis of flavonoids in cells exposed to heavy metals is one strategy that plants employ to resist oxidative stress and counteract toxic effects (Michalak, 2006; Bhattacharya et al., 2010; Okem et al., 2015; Huang et al., 2023). Some studies indicate that Cd generally stimulates flavonoid accumulation by inducing oxidative stress in various plants, such as *Robinia pseudoacacia* L. and *Triticum aestivum* L. (Okem et al., 2015; Jia et al., 2016; Ding et al., 2024). However, the inhibition of flavonoid synthesis by Cd has also been observed (Huang et al., 2023). Overall, Cd significantly affects flavonoid accumulation across many plants, indicating that it may influence the medicinal quality and safety of these plants. Nevertheless, there is limited information regarding the response of flavonoid synthesis in medicinal plants to Cd, particularly in AR. Considering the prevalence of Cd-contaminated soils and the medicinal efficacy of AR, exploring methods to regulate flavonoid accumulation under Cd exposure is crucial for ensuring the quality and safety of AR. Some studies have reported that AR thrives at Cd concentrations in soils ranging from 1 mg kg^−1^ to 5 mg kg^−1^ (Duo et al., 2024; Li et al., 2024), which may pose a potential risk for using AR as medicine.

Due to their ability to form a reciprocal symbiotic relationship with the roots of the majority of plants through mycelium and vesicles approximately 30 d after inoculation (Lee et al., 2018; Wang et al., 2020; Liu et al., 2023), arbuscular mycorrhizal fungi (AMF) have garnered considerable attention. Numerous studies have shown that AMF can significantly enhance the accumulation of flavonoids in various plants by complexing or sequestering cadmium (Cd) (Tabrizi et al., 2015; Janeeshma et al., 2022). Our previous research also indicated that AMF colonization resulted in a significant increase in flavonoids in the roots of *R. pseudoacacia* L. and *Medicago sativa* L. (two Cd-tolerant plants) grown in Cd-polluted soils (Huang et al., 2023; Wang et al., 2023b; Ding et al., 2024). However, AMF inhibited flavonoid synthesis in the roots of *Zea mays* L. (Janeeshma et al., 2022). Generally, the regulation of flavonoid accumulation in plants exposed to Cd stress by AMF may be linked to specific strains and plant species. Although Liu et al. (2019) found that *Funneliformis mosseae* (a strain of AMF) noticeably stimulated calycosin-7-glucoside accumulation in AR, the influence of AMF on flavonoid accumulation in AR grown in heavy metal-contaminated soils remains limited. Considering the stimulation of *F. mosseae* on flavonoid synthesis in certain plants under Cd stress and the significant reduction of Cd uptake by AR during AMF colonization (Tabrizi et al., 2015; Liu et al., 2019; Janeeshma et al., 2022; Li et al., 2023; Huang et al., 2023; Ding et al., 2024; Wang et al., 2023b), we hypothesize that AMF colonization in roots may primarily stimulate flavonoid accumulation in AR grown in Cd-polluted soils. The results will provide insights into the role of AMF in improving the quality of medicinal plants in heavy metal scenarios.

## Materials and methods

### Plant seeds, experimental soils, and AMF strains

Seeds of *Astragali Radix* (*Astragalus membranaceus* (Fisch.) Bunge) were provided by Northwest A & F University, China. After surface sterilization with a 10% (w/w) hydrogen peroxide solution for 10 min, the seeds were thoroughly rinsed with deionized water and then soaked in deionized water to germinate on a Petri dish (Zhang et al., 2016; Huang et al., 2019). The soil used in the experiment was contaminated with Cd and was chosen for remediation based on previous studies conducted in our laboratory regarding the effects of Cd on various plants (Jia et al., 2016; Zhao et al., 2016). This soil, collected from the surface layer (0–20 cm) of cultivated land in Central Shaanxi, China (34°16′N, 108°54′E), had been artificially treated with a solution of 3CdSO_4_·8H_2_O, adhering to China’s current environmental quality standards (GB 15618-2018), with further details provided in previous studies (Jia et al., 2016; Zhao et al., 2016). The soil pH was measured using a pH meter after mixing the soil with distilled water without CO_2_ at a 1:2.5 ratio (Li et al., 2013). Total carbon (C) and nitrogen (N) contents in the soil were determined using the Walkley–Black dichromate oxidation method (Nelson and Sommers, 1982) and the semi-micro Kjeldahl method (McGill and Figueiredo, 1993), respectively. Available phosphorus (P) and potassium (K) contents were assessed using standard laboratory methods (Lu, 2000). The total Cd content was measured using graphite furnace atomic absorption spectroscopy (AAS, EWAI AA-7090, Beijing, China). The soil properties were as follows: it was classified as leached brown soil (according to Chinese soil classification); pH was 8.07 ± 0.03; total C content was 13.61 ± 0.40 g kg^−1^; total N content was 1.16 ± 0.03 g kg^−1^; available P and K contents were 10.7 ± 1.21 and 167 ± 5.36 mg kg^−1^, respectively; and total Cd content was 4.42 ± 0.02 mg kg^−1^. The fungal agent, *F. mosseae* strain (Number, BGC NM 04 A), containing spores and hyphae for the experiment, was purchased from the Beijing Academy of Agriculture and Forestry Sciences, China, with a spore density of 50 ± 1 g^−1^ under a × 250 compound light microscope (China, JueqiXSP-02/06).

### Details in experimental design

A two-factor split-zone test was conducted to investigate the effects of AMF and growth time on flavonoid accumulation in AR grown in Cd-polluted soils. The treatments consisted of non-inoculation (NF) and *F. mosseae* inoculation (FM), with three replicates prepared for each treatment. The pot experiment involved pots (20.5 cm in diameter × 14.2 cm in height) uniformly filled with 3.0 kg of soil. Moreover, 20 g and 10 g of *F. mosseae* agents were distributed at depths of 1 cm and 3 cm in the soil, respectively, and the germinated seeds were planted at a depth of 0.8 cm.

All pots were placed in plant incubators with a capacity of 840 L (Percival E-36 L, Iowa, USA). The parameters in the incubators were set according to the growth habits of AR as follows: night and day temperatures were set to 18°C and 24°C, respectively; average humidity was maintained at 75% ± 4%; and average photon flux density and photoperiod were set to 550 μmol m^−2^ s^−1^ and 12 h, respectively. A total of 15 seedlings per pot were kept with a plant spacing of 3.5 cm for the study after emergence. Seedlings were watered with tap water according to the humidity in the incubators, and a 50% Hoagland nutrient solution was provided once a month throughout the experiment. Additionally, fallen leaves and weeds were removed immediately during culturing.

### Sampling

The entire plants and rhizosphere soils were collected using a multi-point mixing method at 60, 90, and 120 d post-emergence in accordance with the developmental period of AR seedlings. The rhizosphere soil was obtained by shaking and brushing the roots, while the roots and shoots were collected separately. Plant samples were divided into two parts: one was stored at −80°C to assess the relative expression of chalcone synthase (*CHS*) and phenylalanine ammonia-lyase (*PAL*) genes, while the other was dried at 65°C until it reached a constant weight after being heated at 95°C for 3 min to deactivate the enzymes. The dried plant samples were further divided into two portions: one was crushed and sieved through a 0.5-mm sieve for the examination of flavonoids and biomass, while the second was crushed and then sieved through a 0.074-mm sieve to determine the contents of C, N, S, P, H, and Cd. Additionally, the roots were analyzed for biomass and the *F. mosseae* colonization rate, while the rhizosphere soils were used to determine total Cd.

### Determination of plant growth parameters, the *colonization rate* of *F. mosseae,* and Cd levels in rhizosphere soils and plants

Plant height and root length were measured using a ruler with an accuracy of 0.1 cm. Shoot and root biomass were measured using the dry weight method with a balance with an accuracy of 0.1 mg (Mettler Toledo AL204, Switzerland). The P content in plants was analyzed according to the method described by Zavalloni et al. (2012). An elemental analyzer (Elementar, Germany, Vario Macro cube) detected the presence of C, N, S, and H in plants after 15 mg of plant powder was packed in tin foil. The roots for examining *F. mosseae* colonization rates were treated using the method from Zhang et al. (2023). The treated root segments were examined to determine the *F. mosseae* colonization rate under a compound light microscope (× 250) (JueqiXSP-02/06, China) after staining with the ink and vinegar method (Vierheilig et al., 1998). The *F. mosseae* colonization rate was calculated using the method described by Biermann and Linderman (1981).

A total of 20 mg of rhizosphere soils, roots, and shoots were digested in nitric acid-hydrofluoric acid solutions (v/v, 3:1), nitric acid-hydrogen peroxide solutions (v/v, 7:2), and nitric acid-hydrogen peroxide solutions (v/v, 8:3) using a microwave digestion instrument (Shanghai Yiyao, China, WX-6000). Cadmium in the digested solutions was detected using AAS, according to Huang et al. (2023).

### Determination of flavonoids in shoots and roots

Previous studies have indicated that the flavonoids in AR mainly contain calycosin, calycosin-7-glucoside, formononetin, and ononin (Li et al., 2022; Li et al., 2024). Therefore, these four flavonoid monomers were analyzed in this study.

Shoot and root powders were extracted using 70% (v/v) ethanol by refluxing for 3 h according to a solid–liquid ratio of 1:50. The extracts were employed to detect total flavonoids through the aluminum nitrate colorimetric method (Zhang et al., 2008). The contents of calycosin, calycosin-7-glucoside, formononetin, and ononin in the extracts were determined using high-performance liquid chromatography (HPLC, LC-2030C 3D Plus, Shimadzu, Japan) after lipids and pigments were removed with petroleum ether. A Shim-pack GIST C18 column (5 μm, φ4.6 mm × 250 mm, Shimadzu, Japan) served as the stationary phase, while acetonitrile and distilled water were used as the mobile phase. Gradient elution was applied to separate flavonoid monomers, beginning with 20% acetonitrile at 0 min, 30% acetonitrile at 8 min, 43% acetonitrile at 15 min, and 60% acetonitrile at 30 min, respectively. The injection volume was 10 μL, the flow rate of the mobile phases was 1.0 mL min^−1^, the column temperature was maintained at 30°C, and a diode array detector was used to detect flavonoid monomers at 254 nm.

### Analysis of the relative expression of *PAL* and *CHS* genes

Total RNA was extracted from 0.1 g of fresh material using the Biospin Plant Total RNA Extraction Kit (DNA-free) (Bioer Technology, Hangzhou, China). An ultra-micro spectrophotometer (DS-11, DeNovix, USA) assessed the concentration and purity of the RNA. The RNA was used for the reverse transcription reaction when the OD_260_/OD_280_ value ranged from 1.8 to 2.0. cDNA was synthesized with the first strand cDNA synthesis kit (Biosharp, Beijing, China). The quality and concentration of the cDNA were evaluated using an ultra-microspectrophotometer (DS-11, DeNovix, USA). Reverse transcription-quantitative polymerase chain reaction (RT-qPCR) was conducted to measure the relative expression levels of the *PAL* and *CHS* genes using the LightCycler® 480 II thermocycler system (Roche, Switzerland). Primers for the *PAL*, *CHS*, and actin genes were chosen based on the study of Zhang et al. (2023) for RT-qPCR (Jiao et al., 2015). The actin gene (JQ028730.1), known for its stable expression across various tissues and cell types under differing conditions, served to normalize the relative expression of the *PAL* and *CHS* genes (Rahman et al., 2020; Gao et al., 2023). The *PAL* and *CHS* genes were amplified using a touchdown procedure according to the program outlined by Zhang et al. (2023). The specificity of PCR amplification was analyzed using a heat melting curve. Three technical and biological replicates were prepared for all samples. The 2^-ΔΔC^_T_ method was employed to calculate the relative transcript quantity (Livak and Schmittgen, 2001; Zhang et al., 2023).

### Data analysis

SPSS 26.0 software was used for statistical analysis. A two-way analysis of variance was conducted to analyze the effects of *F. mosseae* and time on plant growth, soil characteristics, and Cd in plants and soils. Duncan’s test was carried out at *p* < 0.05 to evaluate the differences between treatments when the difference in the F ratio was significant at *p* < 0.05. Additionally, redundancy analysis (RDA) was performed to evaluate the effect of plant growth characteristics on variations in flavonoids using Canoco 5.0. All data were expressed as mean ± standard error (n = 6).

## Results

### *F. mosseae* colonization scenarios and the colonization rate

**Figure 1:**
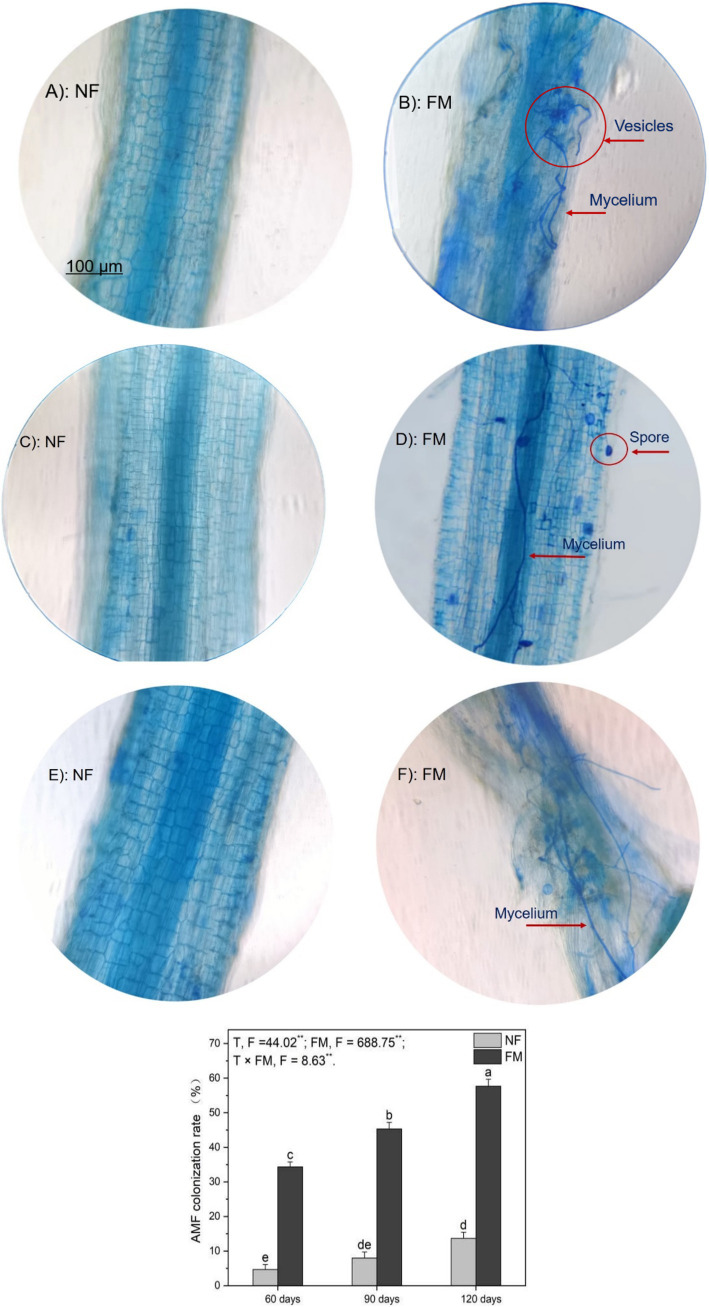
The colonization scenarios and rate of *Funneliformis mosseae* in *Astragali Radix* roots under a 250 × compound light microscope were analyzed using two-way variance analysis (ANOVA), along with a summary of the ANOVA results (*F* values and significance levels). Results are expressed as means ± SE (n = 6). Pictures **(A,C,E)** represent non-inoculation scenarios at 60, 90, and 120 d, respectively, while pictures **(B,D,F)** represent AMF colonization scenarios at 60, 90, and 120 d, respectively. The NF and FM indicate the non-inoculation and *F. mosseae* colonization, respectively. Different lowercase letters indicate significant differences (*p* < 0.05) between different treatments. The ** and * denote significance at *p* < 0.01 and *p* < 0.05, respectively. Ns indicate non-significance.

*F. mosseae* colonization scenarios, including mycelium, spores, and vesicles in AR roots, were clearly observed at 60, 90, and 120 d under inoculation groups (Figure 1). The colonization rate increased significantly over time, with the highest rate of increase (1135.7%) occurring at 120 d under *F. mosseae* inoculation compared to the control (the non-inoculation at 60 d) (Figure 1). However, the rate of increase in the colonization rate decreased over time relative to the non-inoculation (Figure 1). The colonization rate was positively (*p* < 0.05) correlated with root length and negatively (*p* < 0.05) correlated with total Cd in rhizosphere soils (Table 1). Overall, *F. mosseae*, time, and their interaction significantly affected the colonization rate (Figure 1).

**Table 1:** Pearson correlation between *Funneliformis mosseae* colonization rate and biomass, root length, root Cd, and total Cd in rhizosphere soils and in the roots of *Astragali Radix.*

Items	*F. mosseae* colonization rate
Root biomass	0.142
Root length	0.530*
Root Cd	0.449
Total Cd in rhizosphere soils	−0.779**

^**^p < 0.01; ^*^p < 0.05.

### Plant growth characteristics

#### Plant height and root length

**Figure 2:**
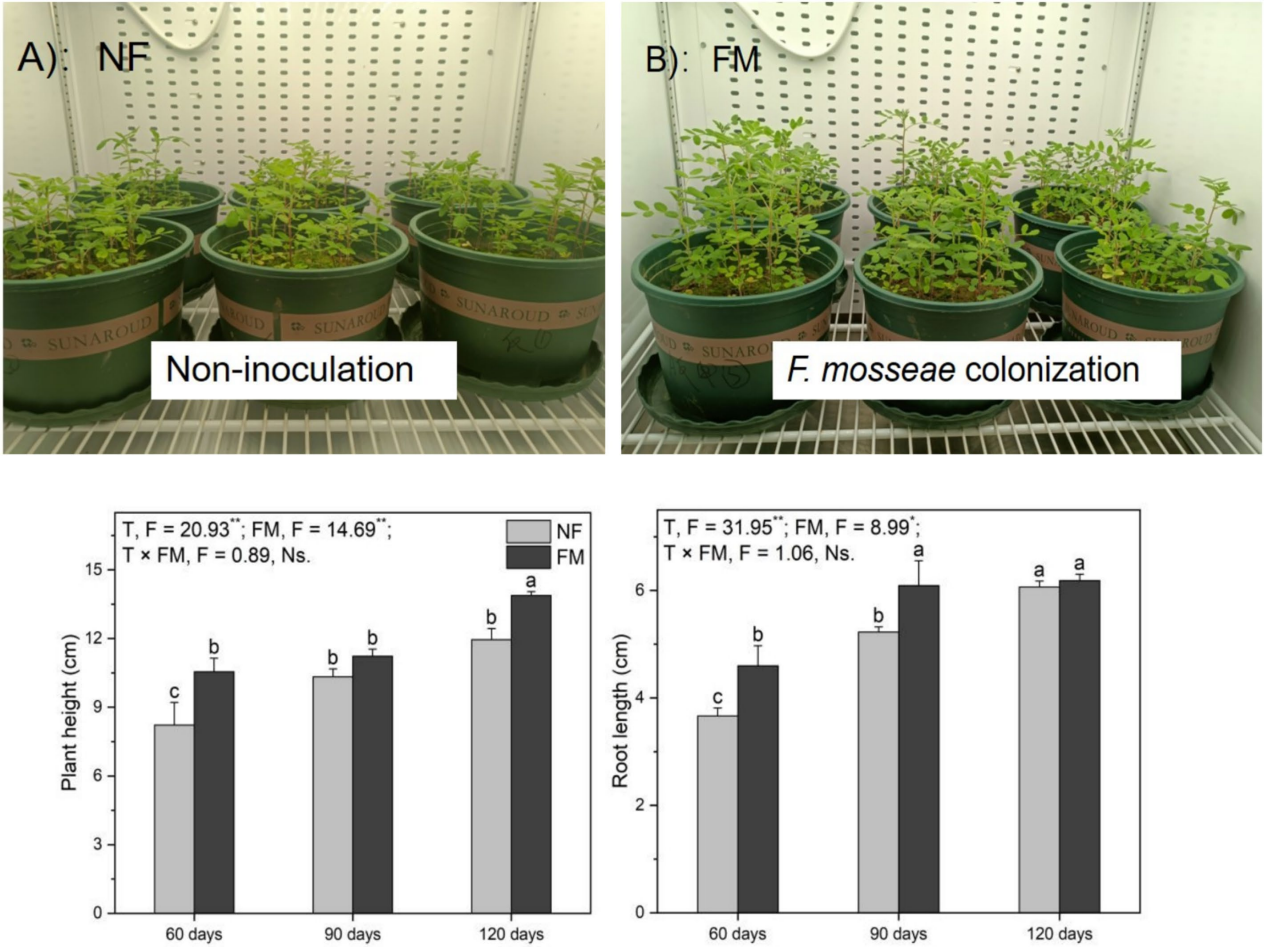
Growth status, height, and root length of *Astragali Radix* seedlings analyzed using two-way variance analysis (ANOVA), along with a summary of the ANOVA results (F values and significance levels). Results are presented as means ± SE (n = 6). Pictures **(A,B)** illustrate *AR* growth status at 60 d, respectively. NF and FM denote non-inoculation and *F. mosseae* colonization, respectively. Different lowercase letters indicate significant differences (*p* < 0.05) among treatments. The ** and * symbols signify significance at *p* < 0.01 and *p* < 0.05, respectively. Ns indicates non-significance.

*F. mosseae* inoculation resulted in a significant increase in AR height at 60 d (Figures 2A,B). Compared to the control, *F. mosseae* inoculation improved (*p* < 0.05) AR height and root length at 90 d (36.6 and 66.2%, respectively) and at 120 d (68.8 and 68.8%, respectively) (Figure 2). *F. mosseae* enhanced (*p* < 0.05) AR height at 60 d (28.3%) and at 120 d (16.1%), as well as root length at 60 d (25.5%) and 90 d (16.6%) relative to the non-inoculation treatment (Figure 2). Root length increased (*p* < 0.05) over time under the non-inoculation treatment relative to the control, with the highest increase rate (65.6%) observed at 120 d (Figure 2). Overall, *F. mosseae* and time significantly affected plant height and root length (Figure 2).

#### Shoot biomass, P, C, H, N, and S contents, and the C/H and C/N ratio

**Figure 3:**
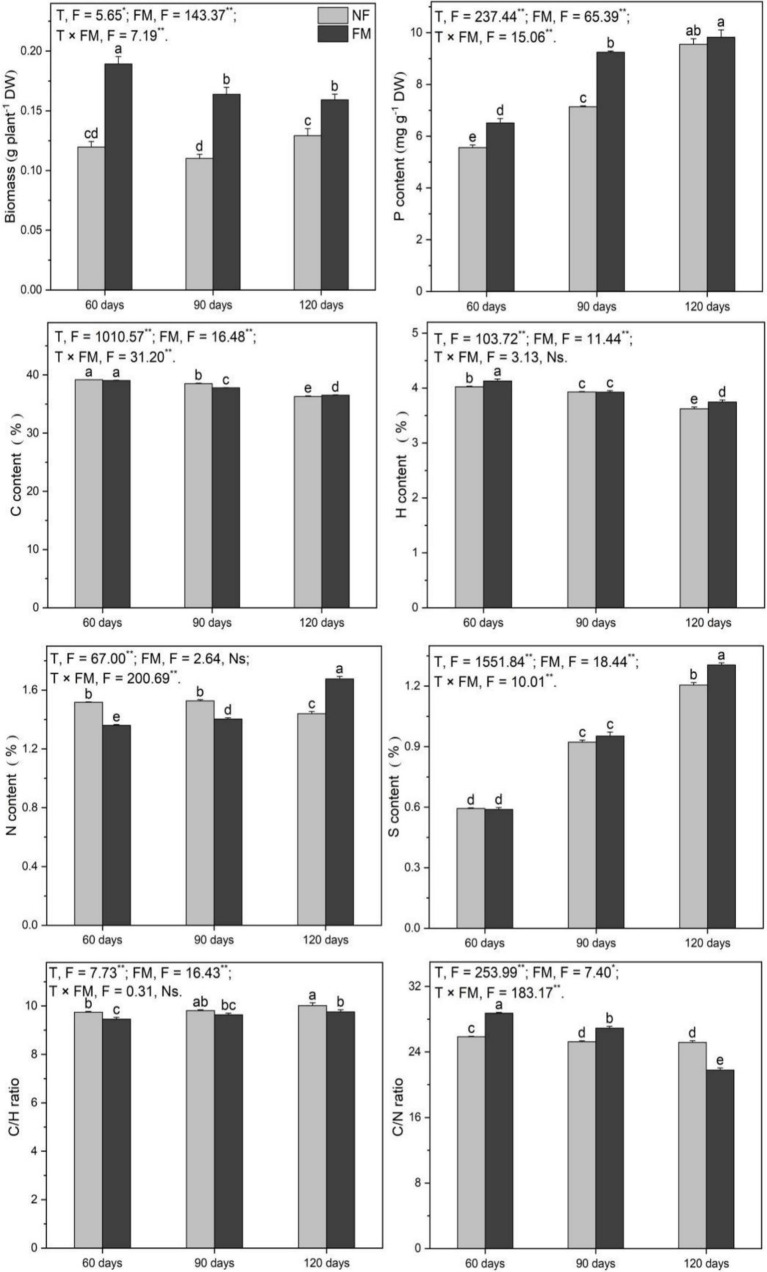
Shoot biomass and the contents of P, C, H, N, and S, along with the C/H and C/N ratios in *Astragali Radix* shoots under various treatments, were analyzed using two-way variance analysis (ANOVA). A summary of the ANOVA results (F values and significance levels) is provided. The results are presented as means ± SE (n = 6). The NF and FM indicate non-inoculation and *F. mosseae* colonization, respectively. Different lowercase letters indicate significant differences (*p* < 0.05) between treatments. The ** and * symbols represent significance at *p* < 0.01 and *p* < 0.05, respectively. Ns indicate non-significance.

*F. mosseae* inoculation stimulated (*p* < 0.05) the biomass at 90 (36.8%) and 120 d (33.0%) relative to the control (Figure 3). The biomass increased (*p* < 0.05) under inoculation compared to non-inoculation, with the highest increase rate (58.0%) at 60 d, and the rate of increase decreased over time (Figure 3).

Shoot P increased (*p* < 0.05) over time, regardless of inoculation, with the highest rate of increase observed at 120 d under non-inoculation (71.8%) and inoculation (76.8%) relative to the control (Figure 3). Additionally, *F. mosseae* inoculation enhanced (*p* < 0.05) P content at 60 d (17.2%) and 90 d (29.5%) relative to non-inoculation, and P increased (*p* < 0.05) by 50.9% at 120 d relative to 60 d under inoculation (Figure 3). The contents of C and H decreased (*p* < 0.05) over time, with the greatest rates of decrease at 120 d under non-inoculation (7.4 and 9.9%, respectively) and inoculation (6.8 and 6.9%, respectively) relative to the control. They also decreased (*p* < 0.05) at 120 d (6.5 and 9.3%, respectively) relative to 60 d under inoculation (Figure 3). Relative to non-inoculation, *F. mosseae* significantly stimulated C and H contents at 120 d, H at 60 d, and reduced C at 90 d (Figure 3). Additionally, the C/H ratio decreased (*p* < 0.05) under inoculation (Figure 3).

Shoot N increased significantly over time, with the highest increase rate (23.3%) observed at 120 d compared to 60 d (Figure 3). Relative to the control, N showed a significant decrease at 90 d (7.5%) and an increase at 120 d (10.5%) under inoculation (Figure 3). Additionally, the inoculation significantly reduced N content at 60 d (10.3%) and 90 d (8.1%), while stimulating N content at 120 d (16.4%) compared to the non-inoculated conditions (Figure 3). Overall, the inoculation enhanced the C/N ratio (*p* < 0.05) at 60 and 90 d; however, the C/N ratio significantly decreased at 120 d (Figure 3). Shoot S increased (*p* < 0.05) over time, reaching the highest rate of increase at 120 d under both non-inoculated (103.0%) and inoculated conditions (119.8%) relative to the control. It increased (*p* < 0.05) by 121.6% at 120 d compared to 60 d under inoculation (Figure 3). Additionally, the inoculation promoted (*p* < 0.05) S content by 8.3% at 120 d (Figure 3).

Overall, *F. mosseae*, time, and their interaction significantly affected biomass, P, and S. *F. mosseae* and time had a significant effect on C, H, and the C/H ratio, while *F. mosseae* and the interaction of the two factors showed significant effects on N (Figure 3).

#### Root biomass, P, C, H, N, and S contents, and C/H and C/N ratio

**Figure 4:**
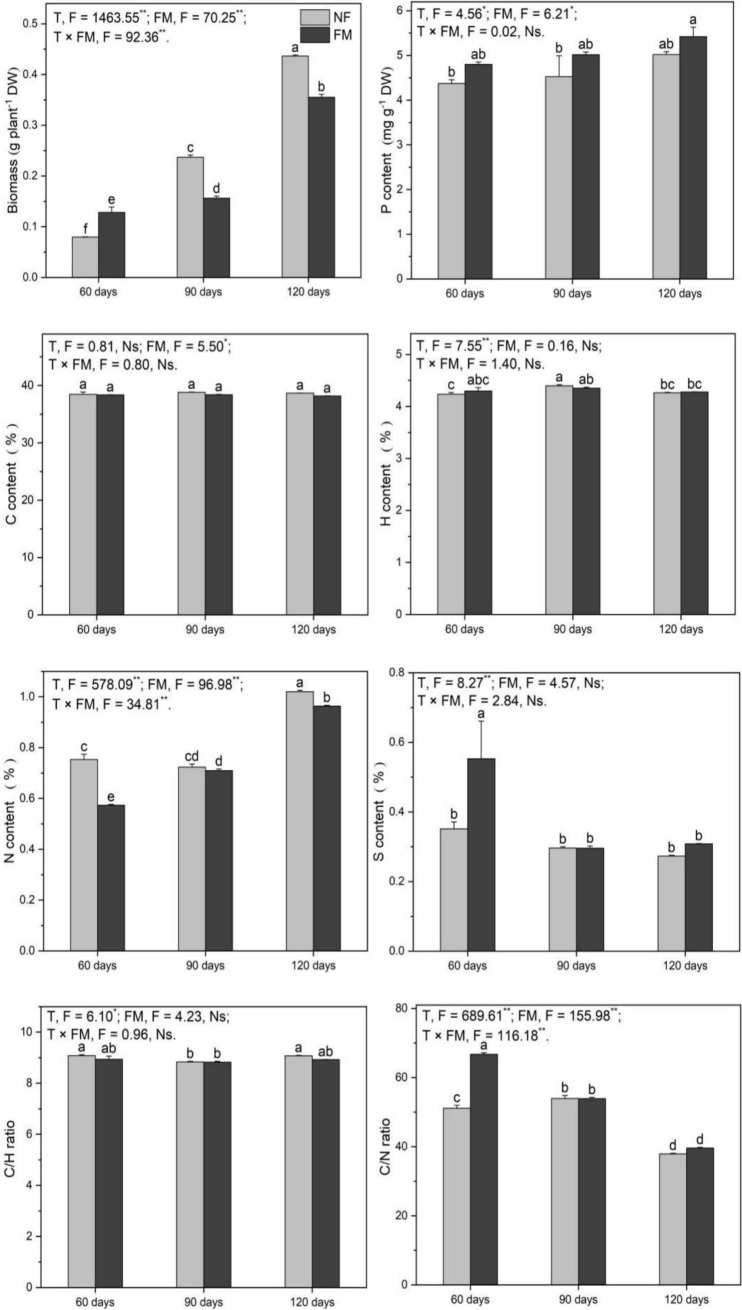
Root biomass and the contents of P, C, H, N, and S, along with C/H and C/N ratios in *Astragali Radix* roots under various treatments, were analyzed using two-way variance analysis (ANOVA). A summary of the ANOVA results (F values and significance levels) is presented. The results are shown as means ± SE (n = 6). The NF and FM represent non-inoculation and *F. mosseae* colonization, respectively. Different lowercase letters indicate significant differences (*p* < 0.05) between different treatments. The ** and * symbols indicate significance at *p* < 0.01 and *p* < 0.05, respectively. Ns indicate non-significance.

Root biomass increased (*p* < 0.05) with time, irrespective of inoculation, with the highest rate of increase at 120 d under both non-inoculation (449.0%) and inoculation (346.8%) relative to the control (Figure 4). Relative to non-inoculation, root biomass significantly increased at 60 d (61.4%) but decreased at 90 d (34.1%) and 120 d (18.6%) under inoculation (Figure 4). Additionally, biomass under inoculation increased (*p* < 0.05) by 176.9% at 120 d relative to 60 d (Figure 4). The inoculation promoted (*p* < 0.05) root P by 23.9% at 120 d relative to the control (Figure 4). Compared with the control, N content decreased (*p* < 0.05) by 5.8% at 90 d and increased by 27.9% at 120 d under inoculation (Figure 4). Root N increased (*p* < 0.05) over time under inoculation, with the highest rate (68.0%) of increase at 120 d relative to 60 d, and decreased (*p* < 0.05) at 60 d (23.9%) and 120 d (5.6%) under inoculation relative to non-inoculation (Figure 4). Additionally, inoculation enhanced (*p* < 0.05) the C/N ratio and S content at 60 d (Figure 4).

Overall, *F. mosseae*, time, and their interaction significantly affected root biomass, N, and the C/N ratio. *F. mosseae* and time significantly affected P, while S was significantly affected by time (Figure 4).

### Cadmium in rhizosphere soils and plants

**Figure 5:**
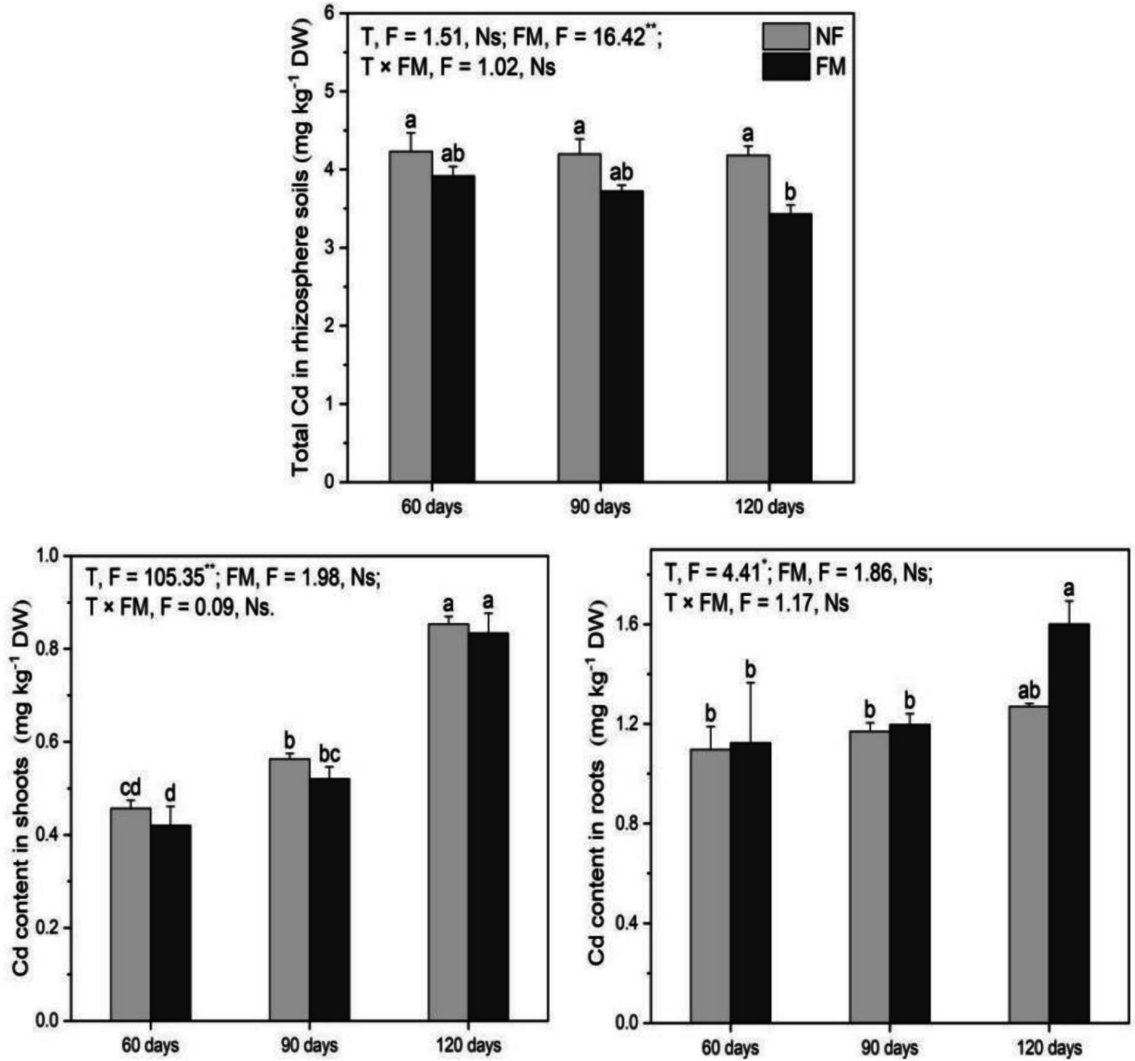
Total Cd contents in rhizosphere soils and in *Astragali Radix* under various treatments were analyzed using two-way variance analysis (ANOVA), accompanied by a summary of the ANOVA results (F values and significance levels). The results are presented as means ± SE (n = 6). The abbreviations NF and FM denote non-inoculation and *F. mosseae* colonization, respectively. Different lowercase letters represent significant differences (*p* < 0.05) among the treatments. The symbols ** and * indicate significance at *p* < 0.01 and *p* < 0.05, respectively, while Ns indicates non-significance.

Total Cd in rhizosphere soils decreased (*p* < 0.05) at 120 d under inoculation relative to the control and non-inoculation by 18.9 and 17.9%, respectively (Figure 5). Shoot Cd increased (*p* < 0.05) with time, with the highest rate of increase at 120 d under non-inoculation (86.9%) and inoculation (82.5%) relative to the control. It increased (*p* < 0.05) by 98.4% at 120 d relative to 60 d under colonization (Figure 5). The inoculation promoted (*p* < 0.05) root Cd by 45.9% at 120 d relative to the control (Figure 5). Overall, *F. mosseae* had a significant effect on total Cd in rhizosphere soils, and time significantly affected shoot and root Cd (Figure 5).

### Flavonoids in plants

#### Flavonoids in shoots

**Figure 6:**
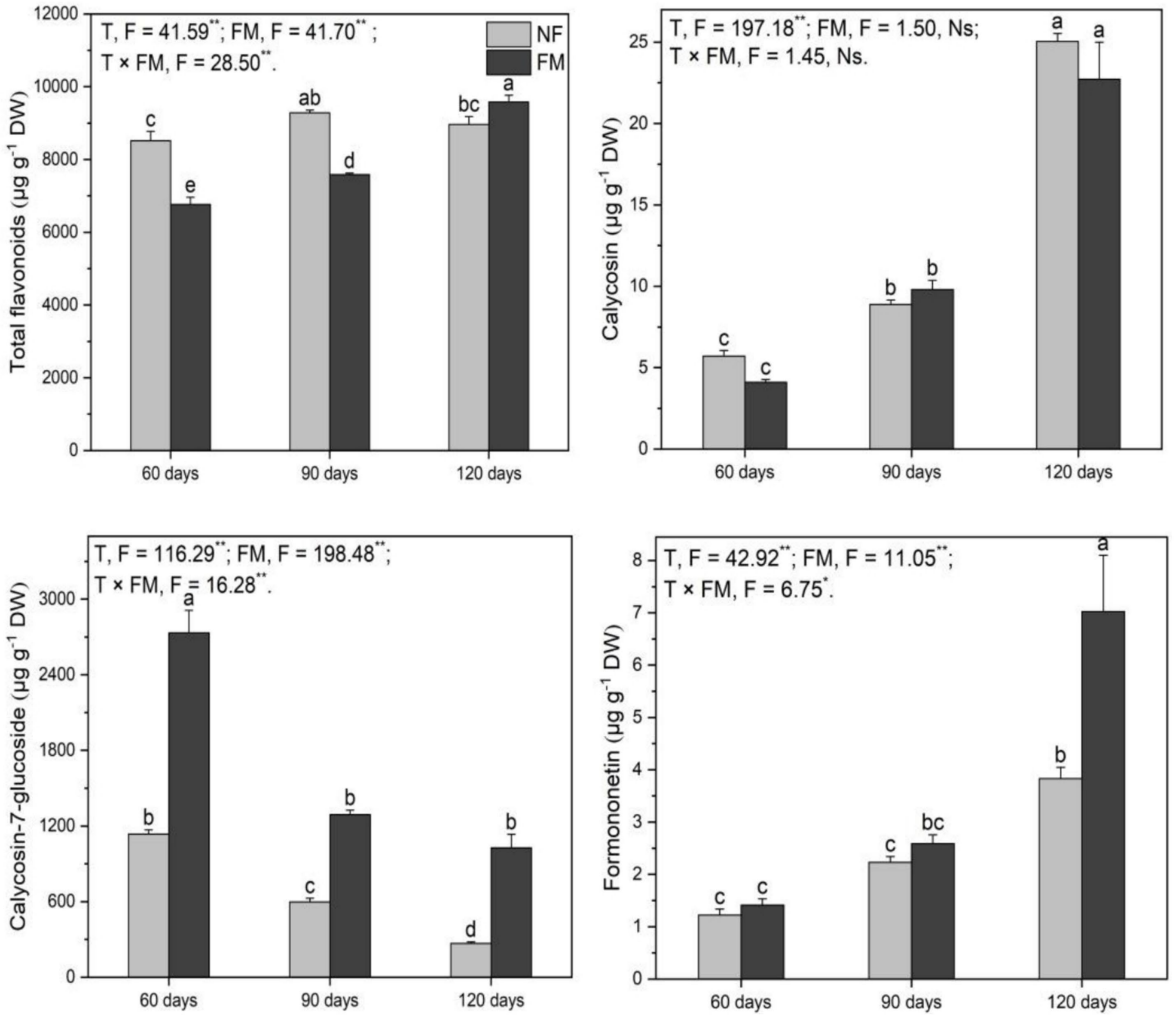
The contents of total flavonoids and monomers in the shoots of *Astragali Radix* under various treatments were analyzed using two-way variance analysis (ANOVA), along with a summary of the ANOVA results, including F values and significance levels. Results are presented as means ± SE (n = 6). NF and FM indicate non-inoculation and *F. mosseae* colonization, respectively. Different lowercase letters denote significant differences (*p* < 0.05) between treatments. The symbols ** and * indicate significance at *p* < 0.01 and *p* < 0.05, respectively, while Ns denotes non-significance.

Compared to the control, the total flavonoid content decreased (*p* < 0.05) at 90 d (11.0%) and increased (*p* < 0.05) at 120 d (12.5%) under inoculation (Figure 6). The total flavonoids increased (*p* < 0.05) over time with inoculation, showing the highest rate of increase at 41.6% at 120 d relative to 60 d (Figure 6). *F. mosseae* reduced (*p* < 0.05) total flavonoids at 60 d (20.5%) and 90 d (18.3%) but enhanced (*p* < 0.05) total flavonoids by 6.9% at 120 d relative to non-inoculation (Figure 6).

Calycosin content increased (*p* < 0.05) over time, regardless of inoculation, with the highest rates of increase at 339.1 and 298.4% at 120 d under non-inoculation and inoculation, respectively, compared to the control. Additionally, it increased (*p* < 0.05) by 452.8% at 120 d relative to 60 d under inoculation (Figure 6). Calycosin-7-glucoside decreased (*p* < 0.05) over time under non-inoculation, showing the highest rate of decrease at 76.4% at 120 d relative to the control (Figure 6). Under inoculation, calycosin-7-glucoside content increased (*p* < 0.05) at 60 (140.8%), 90 (116.4%), and 120 d (283.0%) relative to non-inoculation (Figure 6). Formononetin content increased (*p* < 0.05) by 474.2% at 120 d under inoculation compared to the control (Figure 6). *F. mosseae* resulted in an increase in formononetin of 83.4% at 120 d relative to non-inoculation (Figure 6).

Overall, *F. mosseae*, time, and their interactions significantly affected the total flavonoids, calycosin-7-glucoside, and formononetin, while time also significantly affected calycosin (Figure 6).

#### Flavonoids in roots

**Figure 7:**
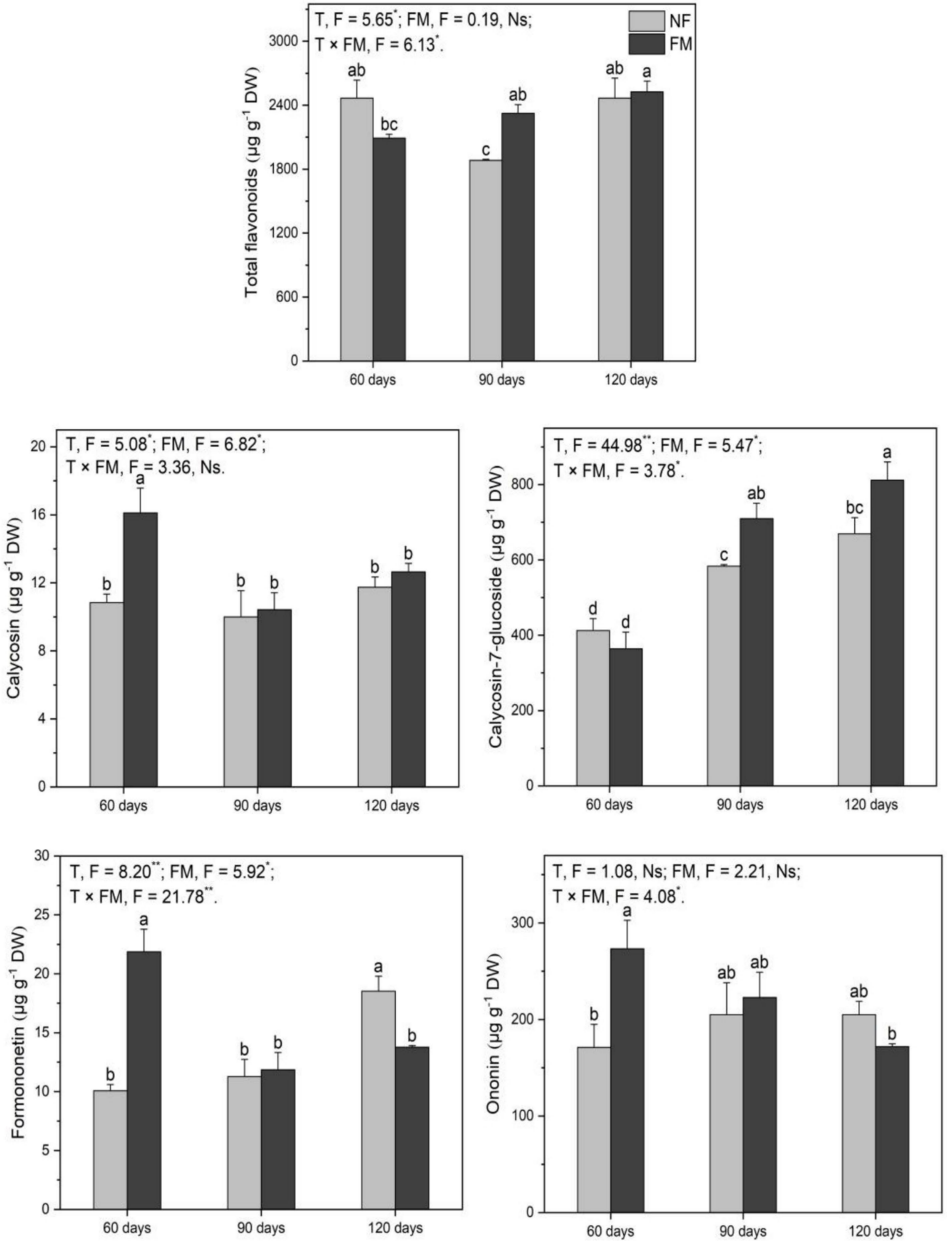
The contents of total flavonoids and monomers in the roots of *Astragali Radix* under various treatments, analyzed using two-way variance analysis (ANOVA), along with a summary of the ANOVA results (F values and significance levels). Results are expressed as means ± SE (n = 6). NF and FM represent non-inoculation and *F. mosseae* colonization, respectively. Different lowercase letters indicate significant differences (*p* < 0.05) among the treatments. The ** and * denote significance at *p* < 0.01 and *p* < 0.05, respectively. Ns indicates non-significance.

*F. mosseae* caused a decrease (*p* < 0.05) in total flavonoids at 60 d (15.2%) and an increase (*p* < 0.05) at 90 d (23.5%) (Figure 7). Under inoculation compared with the control, calycosin-7-glucoside increased (*p* < 0.05) at 90 (72.0%) and 120 d (96.6%), respectively (Figure 7). Calycosin-7-glucoside also increased (*p* < 0.05) at 90 (21.7%) and 120 d (21.2%) under inoculation relative to non-inoculation (Figure 7). Additionally, *F. mosseae* stimulated (*p* < 0.05) calycosin (48.7%), formononetin (117.1%), and ononin (59.6%) at 60 d, while leading to a decrease in (*p* < 0.05) formononetin (25.6%) at 120 d relative to non-inoculation (Figure 7). Overall, the time and the interaction of the two factors significantly affected total flavonoids, while time and *F. mosseae* had significant effects on calycosin. Time, *F. mosseae,* and their interactions showed significant effects on calycosin-7-glucoside and formononetin, and the interaction of the two factors significantly influenced ononin (Figure 7).

### Relative expression of *PAL* and *CHS* genes

**Figure 8:**
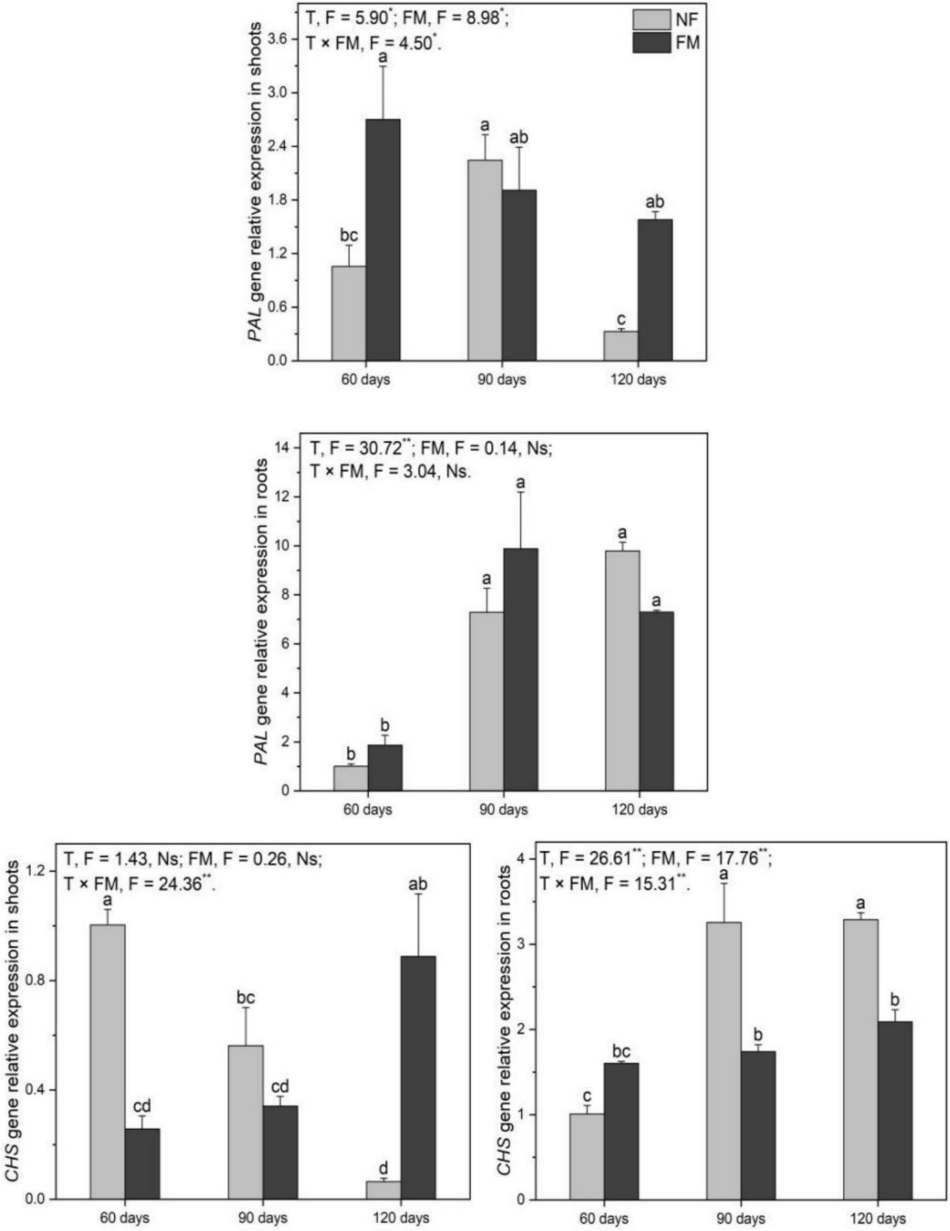
The relative expression of phenylalanine ammonia-lyase (*PAL*) and chalcone synthase (*CHS*) genes in *Astragali Radix* under different treatments was analyzed using two-way variance analysis (ANOVA), along with a summary of the ANOVA results (F values and significance levels). Results are presented as means ± SE (n = 6). NF and FM represent non-inoculation and *F. mosseae* colonization, respectively. Different lowercase letters indicate significant differences (*p* < 0.05) between treatments. The ** and * indicate significance at *p* < 0.01 and *p* < 0.05, respectively. Ns indicates non-significance.

The relative expression of the shoot *CHS* gene decreased over time (*p* < 0.05), reaching the highest rate of downregulation (93.6%) at 120 d under non-inoculation relative to the control (Figure 8). In contrast, inoculation resulted in an upregulation of the shoot *PAL* gene at 60 d (155.9%) and 120 d (382.4%) relative to the non-inoculation group (Figure 8). The relative expression of the shoot *CHS* gene was significantly downregulated (*p* < 0.05) at 90 d, regardless of inoculation, compared to the control (Figure 8). Additionally, the relative expression of the shoot *CHS* gene was significantly downregulated at 60 d (74.4%) and upregulated at 120 d (282.9%) relative to non-inoculation (Figure 8). Compared to the control, the relative expression of the root *PAL* gene was upregulated (*p* < 0.05) at both 90 and 120 d, irrespective of inoculation (Figure 8). The root *CHS* gene under inoculation showed an upregulation (*p* < 0.05) at 90 d (72.4%) and 120 d (107.0%) relative to the control, while it was downregulated (*p* < 0.05) at 90 d (46.6%) and 120 d (36.5%) relative to non-inoculation (Figure 8). Overall, time significantly affected the root *PAL* gene, while time, *F. mosseae,* and their interactions significantly affected the shoot *PAL* and root *CHS* genes; the interactions affecting the shoot *CHS* gene were also significant (Figure 8).

### Factors affecting flavonoid variation in plants

Calycosin-7-glucoside in the shoots and roots was positively significantly correlated with *PAL* gene expression, whereas calycosin in the shoots was negatively correlated with *PAL* gene expression (*p* < 0.05, Table 2).

**Table 2:** Pearson correlation between flavonoids and the relative expression of phenylalanine ammonia-lyase (*PAL*) and chalcone synthase (*CHS*) genes in *Astragali Radix*.

Items		Shoots		Roots	
		*PAL*	*CHS*	*PAL*	*CHS*
Shoots	Total flavonoids	−0.432	0.410		
	Calycosin	−0.559^*^	−0.185		
	Calycosin-7-glucoside	0.589^*^	−0.037		
	Formononetin	−0.231	0.095		
	Ononin	--	--		
Roots	Total flavonoids			0.127	−0.208
	Calycosin			−0.325	−0.106
	Calycosin-7-glucoside			0.787**	0.392
	Formononetin			−0.047	0.189
	Ononin			−0.022	0.087

^**^p < 0.01; ^*^p < 0.05.

**Figure 9:**
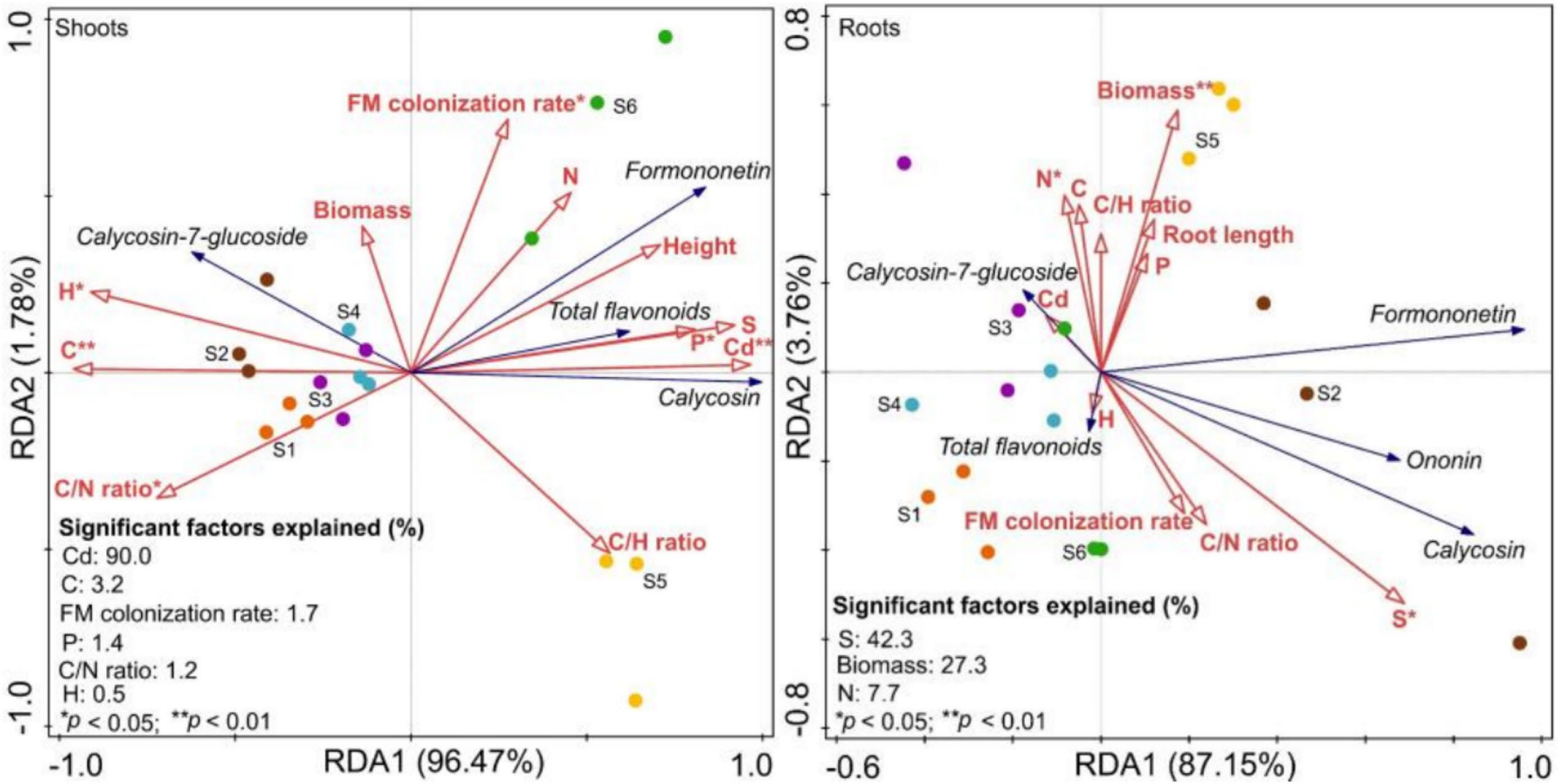
Redundancy analysis (RDA) between flavonoids and growth parameters of *Astragali Radix* seedlings (n = 6). S1 to S3 correspond to non-inoculation at 60, 90, and 120 d, respectively, while S4 to S6 correspond to *F. mosseae* inoculation at 60, 90, and 120 d, respectively.

Plant growth explained 98.3 and 90.9% of the variation in shoot and root flavonoids, respectively (Figure 9). The colonization rate, along with shoot Cd, C, P, H, and C/N ratio, significantly affected flavonoid levels, with the explanation ratio for Cd being the highest (Figure 9). Additionally, total flavonoids, calycosin, and formononetin were positively correlated with shoot Cd, while calycosin-7-glucoside was negatively correlated with shoot Cd (Figure 9). Root biomass, S, and N also significantly affected root flavonoids, with the explanation ratio for S being the highest (Figure 9). Additionally, total flavonoids, calycosin, formononetin, and ononin were positively correlated with root S (Figure 9).

## Discussion

### Effect of *F. mosseae* on flavonoids in AR shoots

Although Cd is precipitated in alkaline soils, the precipitated Cd in rhizosphere soils can be activated by organic acids such as tartaric acid and maleic acid, which are released by AR roots (Zhou et al., 2024), resulting in Cd uptake by AR regardless of inoculation in our study. However, the insignificant change in total Cd in rhizosphere soils may be associated with the sampling method used for these soils. The rhizosphere soils collected by shaking and brushing the roots may contain some bulk soils, which could explain the insignificant change in total Cd. The higher Cd content in roots relative to shoots indicates that Cd primarily exists in the roots. Many studies have shown that AMF inoculation can significantly reduce the transport of Cd from roots to shoots due to sequestration by mycelium, spores, and vesicles (Wang et al., 2020; Xiao et al., 2020), suggesting that the higher Cd in AR roots may mainly reside in the mycelium and vesicles of *F. mosseae*. This indicates that colonization is beneficial for shoot growth under Cd exposure, as evidenced by increased biomass, height, and P content. Thus, the responses of shoot Cd and growth to *F. mosseae* led to variations in flavonoids.

The decreased total flavonoids at 90 d under inoculation compared with the control were inconsistent with our hypothesis, which was associated with lower N according to RDA data. It is well known that N is essential for the secondary metabolism of plants because N deficiency can inhibit or reduce the synthesis of precursors of secondary metabolites by decreasing the production of necessary enzymes in the biosynthesis pathways (Zhang et al., 2017; Zhang et al., 2023), which might explain the reduction in total flavonoids at 90 d. Additionally, the increased total flavonoids at 120 d under inoculation relative to the control were consistent with our hypothesis, which was related to Cd in shoots. Pandey et al. (2023) found that Cd in soils enhanced the accumulation of secondary metabolites in some medicinal plants, such as *Withania somnifera* (L.) Dunal and *Gynura procumbens* (Lour.) Merr., by reviewing extensive literature, suggesting that Cd could promote flavonoid synthesis in plants. Thus, an increase in shoot Cd at 120 d promoted the production of total flavonoids. Similarly, the increased total flavonoids at 120 d under inoculation relative to the non-inoculated group were consistent with our hypothesis, which might be associated with an increase in shoot N. The increased N might lead to higher expression of the *CHS* gene due to sufficient nitrogen supply, which enhances total flavonoid content. Similarly, the decreased total flavonoids at 60 and 90 d under inoculation relative to the non-inoculated group might be associated with lower N. Additionally, the increased total flavonoids over time under inoculation were distinctly influenced by Cd and P, according to RDA data. Furthermore, the total flavonoids, Cd, and P contents simultaneously reached the highest rate of increase at 120 d, suggesting that P and Cd might stimulate flavonoid synthesis. Some studies indicated that Cd could induce plants to synthesize more flavonoids (Verma and Shukla, 2015; Zhang et al., 2023), which is consistent with our results. As an essential element for energy supply and active centers of enzymes for plant growth, P is involved in photosynthesis and secondary metabolite synthesis (Bhantana et al., 2021). Additionally, Fan et al. (2023) found that P promoted flavonoid synthesis in *Dendrobium*. Thus, the increased P content over time caused by *F. mosseae* could provide more adenosine triphosphate and enzymes for flavonoid synthesis, leading to increased total flavonoids.

Ononin was not detected in shoots, possibly due to the tissue-specific nature of flavonoids, as reported by Dixon and Paiva (1995). Additionally, ononin content may have been too low to reach the detection limit of HPLC. Overall, the effect of *F. mosseae* on the three monomers was different, with increased calycosin at 90 and 120 d and formononetin at 120 d under inoculation compared to the control, which may be associated with higher Cd and P according to RDA data. The stimulation of *F. mosseae* on calycosin-7-glucoside and formononetin was consistent with our hypothesis and was associated with Cd, C, and P contents, as well as the *F. mosseae* colonization rate according to RDA data. Therefore, the increased levels of calycosin-7-glucoside and formononetin under inoculation, compared to non-inoculation, may result from a higher *F. mosseae* colonization rate. Some studies have indicated that AMF stimulated flavonoid synthesis in various plants, such as *Glycyrrhiza uralensis* Fisch. and *Calendula officinalis* L. (Chen et al., 2017; Kheyri et al., 2022), which supports our findings. Additionally, the increase in calycosin-7-glucoside was primarily driven by greater expression of the *PAL* gene, according to Pearson correlation analysis. As a key enzyme in flavonoid synthesis (Guo et al., 2024), PAL activity might increase with higher PAL gene *expression*, which could enhance calycosin-7-glucoside synthesis. Additionally, the increased calycosin-7-glucoside under inoculation relative to non-inoculation was affected by C and the C/N ratio according to RDA data, consistent with the findings reported by Zhang et al. (2023). The higher C/N ratio at 60 and 90 d under inoculation relative to non-inoculation indicates that *F. mosseae* enhanced carbon retention in AR, which might provide more precursors for calycosin-7-glucoside synthesis. Similarly, the increased calycosin-7-glucoside at 120 d might be linked to higher C levels in shoots; this increase suggests that *F. mosseae’s* stimulation of AR photosynthesis could promote calycosin-7-glucoside synthesis. Additionally, the significant rise in calycosin and formononetin over time, regardless of inoculation, was distinctly influenced by Cd, according to RDA data. Many studies have indicated that Cd is a clear inducer of flavonoid synthesis in plants (Verma and Shukla, 2015; Fan et al., 2023), which supports our results. However, the significant decrease in calycosin-7-glucoside over time under non-inoculation was distinctly influenced by shoot C and H, according to RDA data. Since C and H contents in plants can reflect the abundance of substrates for secondary metabolite synthesis, lower C and H contents may explain the decrease in calycosin-7-glucoside. Overall, calycosin-7-glucoside was the most abundant flavonoid detected in shoots.

### Effect of *F. mosseae* on flavonoids in AR roots

Previous studies showed that AMF stimulated or inhibited flavonoid synthesis in some plants, such as red sage, licorice, and black locust (Chen et al., 2017; Wu et al., 2021; Zhang et al., 2023), which suggested that the regulation of AMF on flavonoid synthesis might be related to plant species. Thus, the inoculation caused a decrease in the total flavonoids at 60 d; however, the increase in total flavonoids at 90 d suggested that the effect of *F. mosseae* on flavonoid synthesis might also be affected by growth time. Growth time led to higher root biomass and N and S contents under *F. mosseae* inoculation, which might provide abundant precursors for flavonoid synthesis, resulting in higher flavonoids at 90 d than at 60 d. Additionally, the decreased total flavonoids at 60 d under inoculation, compared with non-inoculation, were associated with S variation in plants according to RDA data, which was inconsistent with our hypothesis. Zhang et al. (2020) found that S deficiency induced flavonoid synthesis in AR; thus, the decreased total flavonoids at 60 d under inoculation were associated with increased root S, according to RDA data. The stimulation of inoculation on total flavonoids at 90 d was associated with greater root biomass, according to RDA data. The increased biomass might provide abundant precursors for synthesizing secondary metabolites, which could result in an increase in total flavonoids. Zhang et al. (2023) found that black locust biomass significantly affected flavonoid monomer synthesis, which supported our results.

The stimulation of *F. mosseae* on calycosin-7-glucoside in roots at 90 and 120 d post-inoculation, compared to the control, was consistent with our hypothesis and correlated with greater biomass and *PAL* gene expression, according to RDA and Pearson correlation data. Additionally, as a key gene in flavonoid synthesis pathways (Zang et al., 2015), the *PAL* gene may promote the synthesis of calycosin-7-glucoside due to the upregulation of its relative expression. Stewart et al. (2001) found that low N levels promoted flavonoid synthesis; thus, the increased calycosin-7-glucoside at 90 and 120 d post-inoculation, relative to the non-inoculated control, might be attributed to N deficiency in the roots. Excess N can enter the secondary metabolism pathway as a raw material for enzymes regulating the synthesis of secondary metabolites after the completion of primary growth and metabolism (Ncube et al., 2012; Jaiswal et al., 2022), suggesting that low N levels may inhibit flavonoid synthesis. Therefore, the decreased formononetin at 120 d post-inoculation, relative to the non-inoculated control, was associated with lower N levels. The increased calycosin, formononetin, and ononin at 60 d post-inoculation, compared to the non-inoculated control, may be related to increased S levels, as indicated by RDA data. Approximately 90% of S is present as methionine and cysteine, which are essential for secondary metabolism in most plants (Matraszek et al., 2016). Thus, the increased S levels in the roots caused by *F. mosseae* led to significant increases in calycosin, formononetin, and ononin. Furthermore, the reduced formononetin at 120 d post-inoculation, compared to the non-inoculated control, was associated with lower biomass.

Overall, calycosin-7-glucoside was the most abundant among the detected flavonoids; however, its accumulation was lower in roots compared to shoots. Moreover, the total flavonoid content was higher in shoots than in roots, suggesting that annual AR shoots may possess significant medicinal value. Additionally, a possible reason for the noticeable increases in calycosin, formononetin, and ononin, observed with the decreased total flavonoids at 60 d under inoculation relative to non-inoculation, may be associated with the inhibition of *F. mosseae* on other flavonoid monomers that could not be detected.

## Conclusion

Overall, calycosin-7-glucoside was the primary flavonoid in annual AR. Additionally, the contents of calycosin-7-glucoside and total flavonoids were higher in the shoots than in the roots, suggesting that the shoots of annual AR possess significant medicinal value. However, the content of formononetin was greater in the roots, and ononin was present in the roots. *Funneliformis mosseae* significantly affected the accumulation of total flavonoids, calycosin-7-glucoside, and formononetin in the shoots of *Astragali Radix* (AR) grown in cadmium (Cd)-contaminated soils. Generally, *F. mosseae* colonization significantly stimulated the accumulation of calycosin-7-glucoside and formononetin in the shoots; however, the levels of calycosin, formononetin, and ononin in the roots increased significantly only at 60 d under *F. mosseae* inoculation. The levels of Cd, C, P, H, and the C/N ratio in the shoots, along with the colonization rate, were significant factors affecting the variation of flavonoids in the shoots. In contrast, root S, N, and biomass significantly affected flavonoid accumulation in the roots. Additionally, the regulation of *F. mosseae* on the expression of the phenylalanine ammonia-lyase gene significantly affected calycosin-7-glucoside synthesis. These results could provide insights into the safety of medicinal plants cultivated in heavy metal-polluted soils. Considering that AMF can mitigate Cd migration from the roots to the leaves by intercepting and chelating heavy metals through mycelium and vesicles in mycorrhizal symbiosis, the study of whether Cd is distributed in AR roots or accumulated by *F. mosseae* will be thoroughly explored to ensure the safety of using the leaves in medicine in the future.

## Data Availability

The original contributions presented in the study are included in the article/supplementary material, further inquiries can be directed to the corresponding author.
